# Metal Chalcogenide–Hydroxide Hybrids as an Emerging Family of Two-Dimensional Heterolayered Materials: An Early Review

**DOI:** 10.3390/ma16196381

**Published:** 2023-09-24

**Authors:** Yuri Mikhlin, Maxim Likhatski, Roman Borisov, Denis Karpov, Sergey Vorobyev

**Affiliations:** 1 Institute of Chemistry and Chemical Technology, Krasnoyarsk Science Center of the Siberian Branch of the Russian Academy of Sciences, Krasnoyarsk 660036, Russia; lixmax@icct.ru (M.L.); roma_boris@list.ru (R.B.); denikarp@mail.ru (D.K.); yekspatz@yandex.ru (S.V.); 2 Department of Chemistry, Bar-Ilan University, Ramat Gan 52900, Israel; 3 Institute of Nonferrous Metals and Materials Science, Siberian Federal University, Krasnoyarsk 660041, Russia

**Keywords:** two-dimensional materials, layered minerals, valleriite, tochilinite, heterostructure, hydrothermal synthesis, Mössbauer spectroscopy, XPS

## Abstract

Two-dimensional (2D) materials and phenomena attract huge attention in modern science. Herein, we introduce a family of layered materials inspired by the minerals valleriite and tochilinite, which are composed of alternating “incompatible”, and often incommensurate, quasi-atomic sheets of transition metal chalcogenide (sulfides and selenides of Fe, Fe-Cu and other metals) and hydroxide of Mg, Al, Fe, Li, etc., stacked via electrostatic interaction rather than van der Waals forces. We survey the data available on the composition and structure of the layered minerals, laboratory syntheses of such materials and the effect of reaction conditions on the phase purity, morphology and composition of the products. The spectroscopic results (Mössbauer, X-ray photoelectron, X-ray absorption, Raman, UV-vis, etc.), physical (electron, magnetic, optical and some others) characteristics, a specificity of thermal behavior of the materials are discussed. The family of superconductors (FeSe)·(Li,Fe)(OH) having a similar layered structure is briefly considered too. Finally, promising research directions and applications of the valleriite-type substances as a new class of prospective multifunctional 2D materials are outlined.

## 1. Introduction

**Figure 1:**
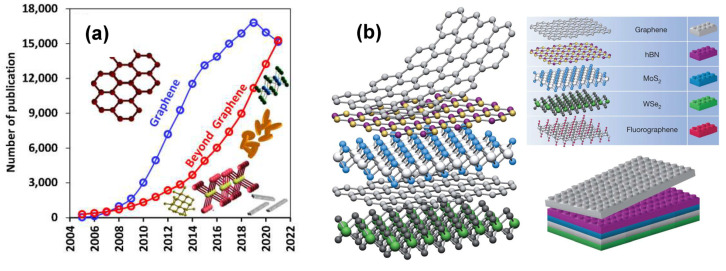
(**a**) Number of publications in the domain of 2D nanomaterials [7] (licensed under CC BY 4.0). (**b**) Scheme of formation of heterostructured 2D materials [35]; permission from Springer Nature.

In this survey, we introduce a group of nature-inspired materials of valleriite, (Fe,Cu)S_2_·*n*(Mg,Fe,Al)(OH)_2_), type, with unusual chemical composition and a structure formed by alternating two-dimensional (2D) heterolayers, which are interesting for plentiful applications. Two-dimensional materials are attracting tremendous attention at present (Figure 1a), owing to their numerous unique properties [1,2,3,4,5,6,7,8,9,10,11,12,13,14,15,16,17,18,19,20]. Graphene is generally considered as the first and most studied 2D material, exhibiting giant electron mobility and conductivity, as well as extraordinary optical, thermal, mechanical and other properties (see, for example, reviews [2,3,4,5,6,7]). Since the seminal study by Geim, Novoselov et al. published in 2004 [1], many different 2D materials have been reported, although some of them were already well-known as natural minerals (molybdenite MoS_2_, clays, layered double hydroxides, etc.) or were synthesized in laboratory. Generally, the density of dangling bonds is very low on the atomic layers and the ones interact via weak van der Waals (vdW) forces. Like graphene, atomically thin layers can be prepared by using exfoliation of bulk materials, and a variety of chemical methods have been developed too. Let us briefly describe several examples of 2D materials, which may be compared with valleriites.

Two-dimensional transition metal dichalcogenides (TMDC) comprise an atomic plane of cations and chalcogen atoms above and below the plane, with the general formula MX_2_ (M is a d-metal, X is S, Se or Te) [10,11,12,13,14,15,16,17]. TMDCs exhibit metallic or semimetallic (VS_2_, NbS_2_, TaS_2_) and semiconducting characteristics (Mo and W sulfides and selenides, TiS_2_, HfS_2_, ZrS_2_, etc.). The direct band gap and excitonic effects arise upon the transition from multi- to monolayer in MoS_2_ and some other TMDCs. This promotes strong optical absorption in the UV-vis-NIR regions in conjunction with rather high electron mobility, which can be controlled by modifying the structure (including the number of stacked layers), and inspires their applications in 2D electronic and optoelectronic devices and photocatalysis [12,13,14,15,16,17]. The chemical and mechanical stability of TMDCs (especially of Mo and W) together with their high surface area make them interesting for tribology, electrode materials for Li ion batteries, (electro)catalysis, and so on. On the other hand, the low concentration of active sites requires additional chemical pre-treatment. To increase the electric conductivity and surface area, TMDCs are often utilized together with graphene and other carbon materials.

**Figure 2:**
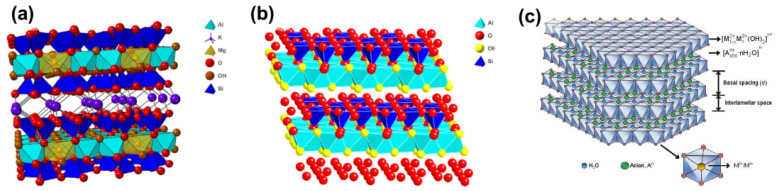
Typical crystal structures of layered clay minerals: (**a**) montmorillonite, a 2:1 type smectite, with layers consisting of two tetrahedral sheets and one octahedral sheet separated by the interlayer space with hydrated cations; (**b**) kaolinite, a 1:1 type clay mineral, with layers consisting of one tetrahedral and one octahedral sheet. Reproduced from Ref. [28] with permission from the Royal Society of Chemistry (RSC). (**c**) Schematic LDH structure [29]. © Distributed by Creative Commons Attribution 3.0 License.

MXenes [18,19,20,21,22,23,24,25] are the family of 2D materials M_n+1_X_n_T_z_ or M_1.33_X_n_T_z_ (where *n* = 1–4; M is a transition metal, X is carbon and/or nitrogen, T is a termination group such as -OH, =O or -F). They have been extensively studied since 2011, when Ti_3_C_2_ single layers were obtained in HF medium [18]. MXenes possess high electric conductivity, strong absorption in the near-IR region, and relatively high chemical resistance. In contrast to graphene and TMDC, -OH or =O terminations make them hydrophilic, extending the range of possible properties and applications, e.g., in sorption, catalysis, electrode materials and biomedicine. Exchanging the metal or specific surface groups allows them to influence electronic, optical (plasmonic) and other characteristics of MXenes. Theoretical calculations suggested that MXenes can be semiconductors, topological insulators and superconductors, for example in case of Nb_2_CT_x_ with Se, S or NH terminations [25]. Nevertheless, preparation of magnetic or semiconducting MXenes, and even uniform surface termination on carbide/nitride MXenes, remains challenging.

(Nano)clay minerals (Figure 2) have a layered structure formed by tetrahedral sheets, in which the silicon–oxygen tetrahedra share three corners, while the fourth is connected with an adjacent octahedral sheet [26,27,28,29,30,31]. The octahedral sheet is typically composed of aluminum and/or magnesium in coordination with hydroxide ions, and with oxygen from the tetrahedra. The layers can have either a negative charge neutralized by interlayer aqueous cations (cationic clays like montmorillonite) or a positive charge balanced by variable anions in the interlayer space (anionic clays, including double-layered hydroxides). Natural clay minerals have been known for centuries, and their synthetic analogs are widely utilized due to their abundance, high specific area, sorption ability and chemical reactivity. Layered double hydroxides (LDHs) [32,33,34] have the common formula M_1−x_^2+^[M_x_^3+^(OH)_2_(A^n−^)_x/n_]^x+^·mH_2_O, where M^2+^ and M^3+^ are cations Mg^2+^, Ca^2+^, Fe^2+^, Fe^3+^, Al^3+^ and others within the positively charged brucite-like layers, and A stands for a variety of interlayered anions (CO_3_^2−^, Cl^−^, NO_3_^−^, SO_4_^2−^, etc.) (Figure 2c). The tunable chemical composition, reversible thermal and pH-sensitive (de)hydroxylation, high ionic 2D conductivity, anion exchange ability and composite formation stimulate the usage of LDHs as adsorbents, catalysts (often after incorporation of modifiers), energy storage materials, carriers for drugs and biological molecules, and many others. However, dielectric properties of LDHs, low stability toward the formation of defects, and non-ordered intercalates restrict their applications as electronic, magnetic and photosensitive materials.

Two-dimensional phenomena take place not only in free-standing monolayers, but also in atomic layers deposited on a support and few-layer materials. A number of bulk properties are actually due to the inner 2D structure as, for example, superconductivity localized in certain crystallographic planes. New remarkable opportunities can arise if two or more different 2D crystals are combined in one vertical stack [35,36,37,38,39,40], producing several thick atomic layer heterostructures (Figure 1b). Arbitrary 2D layers interacting via vdW forces have been predicted theoretically, but only few heterostructures with a small lattice mismatch were stacked mechanically or synthesized by means of atomic layer deposition and similar complicated methods [38]. It is worth recalling that the heterostructures formed by two different (though rather similar, e.g., of A^III^B^VI^ group and quasi-bulk) semiconductors have been widely used in many solid-state devices for decades. So, the development of new prospective 2D heterostructured materials is an important challenge.

Meanwhile, several naturally occurring valleriite-type minerals and synthetic “nature-inspired” metal chalcogenide–hydroxide materials, as well as superconductors composed of FeSe and (Li,Fe)OH molecular layers, are known at present. The layers are probably interacting not via vdW forces but the opposite electric charges; the distances between chalcogenide sheets detached by dielectric hydroxide ones as large as ~11.4 Å suggest a combination of 2D and 3D characteristics. These types of materials are mostly neglected in modern materials science and are restricted by surprisingly narrow considerations in superconductor physics and Earth and meteoritic sciences.

The aim of this article is to summarize the still very limited results on the natural and synthetic heterolayered materials available up to now in order to introduce those to researchers from various fields. First, the minerals of the valleriite family are considered in terms of the chemical composition and crystalline structure; the geological aspects are mainly omitted. A fairly full survey of laboratory syntheses of their analogues is presented. Then, up-to-date studies on the electronic structure and physical and chemical properties are given both for natural and synthetic materials. The superconductors are described concisely; the readers can refer for more detail to the relevant literature. Finally, we outline interesting characteristics of the materials currently found and some promising applications; this part is certainly incomplete, as much more work is required.

## 2. Natural Two-Dimensional Layered Minerals

Valleriite was first discovered by Blomstrand [41] in Sweden more than 150 years ago, but a lot of confusion regarding its composition, structure and discrimination with such minerals as chalcopyrite CuFeS_2_ and mackinawite FeS took place until the 1960s, when Evans and Allmann [42] managed to examine a single crystal of valleriite and establish that its lattice is constructed by alternating quasi-monolayers of Cu-Fe sulfide and brucite (Mg,Al)(OH)_2_. Later, tochilinite FeS·(Mg,Al)(OH)_2_ [43,44,45] and a number of similar heterolayered minerals were identified and their structures confirmed. Below, the terms “vallerite” or “valleriites” will mark both the specific substance formed by Mg-based hydroxide and Cu-Fe-S structural parts and a wider family of hydroxide-chalcogenide materials.

### 2.1. Brucite and Mackinawite

**Figure 3:**
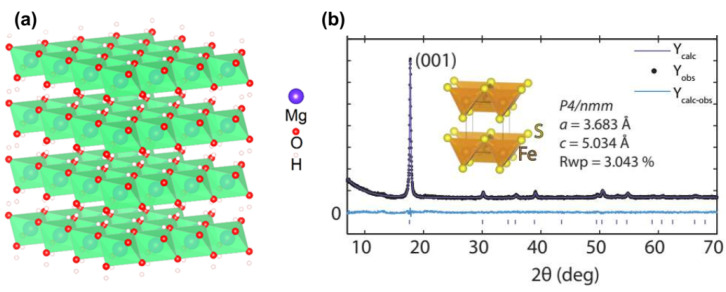
Schematic representation of (**a**) brucite Mg(OH)_2_ structure viewed down the z axis; (**b**) the tetragonal structure and XRD pattern of mackinawite FeS (synthetic). Reproduced from [54] with permission from APS.

Before the discussion of the valleriite-group minerals, let us mention two related compounds. Brucite with the ideal chemical composition Mg(OH)_2_, formed in nature as a low-temperature hydrothermal mineral, is built with a sheet of Mg^2+^ cations between two sheets of hydroxide anions, with each Mg^2+^ being in the center of an octahedron of hydroxyls (Figure 3a) [27]. The layers are composed of the octahedra linked laterally by sharing the edges; the Mg–OH, OH–OH and O–H distances are 2.10 Å, 3.218 Å and 1.03 Å [46]. Important features of brucite are the ability to exchange Mg^2+^ for other cations, including Li^+^, Na^+^, Fe^2+^/Fe^3+^, Al^3+^, Ga^3+^; positive zeta potential up to very high pH; and easy dehydration [27,46,47,48,49].

Mackinawite, a metastable iron sulfide with a tetragonal layered structure [50,51,52,53,54,55], was discovered in Ni-Au ores in Mackinow mine, and first was erroneously identified as valleriite [50]; it also was detected in the products of steel corrosion in the presence of sulfide ions [51]. Mücke [56] specified its composition as (Fe,Ni,Co)_1+x_S (x ranges from −0.10 to +0.10). The crystal structure of mackinawite is constructed [52,54] by edge-connected and edge-supported FeS_4_ tetrahedra (space group P4/nmm) (Figure 3b). The layers are stacked by vdW forces, allowing for the intercalation of various species [57]. Iron cations are in a low-spin singlet Fe^2+^ state but easily transform to high-spin Fe^2+^-S and Fe^3+^-S centers, S-excessive composition and so forth under moderate oxidative conditions [58,59]. Iron selenide β-FeSe with the same tetragonal structure is a superconductor (T_C_~40 K) [60,61,62], and superconducting behavior has been reported for the tetragonal FeS at about 4 K [57].

### 2.2. Valleriites

The chemical composition of natural valleriites notably vary for the samples examined; for example, Evans and Allmann [42] have reported the composition (Fe_1.07_Cu_0.93_) S_2_·1.526[(Mg_0.68_Al_0.32_)(OH)_2_], and Mücke [56] has suggested the formula (Fe,Cu)_2_S_2_·*x*[(Mg^2+^,Fe^2+^,Al^3+^_y_)(OH)_2_], where *x* = 1.24–2.25 and *y* = 0.00–0.26, for valleriites from five different locations. The proportion of the metal sulfide and brucite-like parts is not constant; the hydroxide layers contain variable quantities of Al, Fe and other cations, and there exist minerals with mainly Fe (ferrovalleriite) [63] or Cr (chromian valleriite) [64].

**Figure 4:**
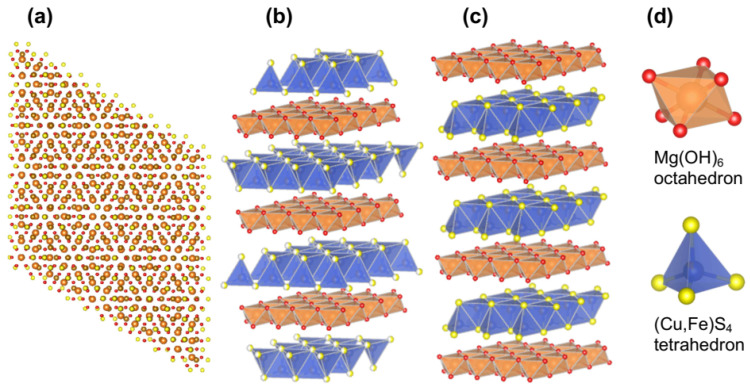
Schemes of crystalline structures of (**a**,**b**) “single-layer” and (**c**) “three-layer” valleriites, and (**d**) coordination units in the hydroxide and sulfide layers.

The crystalline lattice of valleriite [42] is formed by hexagonal brucite-based layers (space group *P*$\bar{3}$*m*1) with the cell parameters *a* = 0.307 nm and *c* = 1.137 nm, and rhombohedral sulfide layers (*R*$\bar{3}$*m*) with *a* = 0.379 nm and *c* = 0.341 nm (Figure 4). The inconsistency of the constant *a* for the sulfide and hydroxide layers causes a moiré picture upon stacking the layers (Figure 4a). In addition to the “three-layer” valleriite, Organova [65,66] identified a “single-layer” variety, in which both the layers are crystalized in a trigonal lattice (space group *P*$\bar{3}$*m*1) with *c* = 1.137 nm. The strongest reflections in the X-ray diffraction patterns at 1.13–1.14 nm and 0.56–0.57 nm are explicit features of valleriite and related compounds. It has been suggested that some diffraction features may be due to ordering Fe cations in the hydroxide layers, but no super-lattices were found [66]. The arrangement of metal and sulfur atoms seems to be similar to that of nukundamite (Cu,Fe)_4_S_4_, a layered sulfide resembling CuS [67,68]. Statistical distribution of Cu and Fe atoms in the sulfide layers has been hypothesized but not confirmed experimentally. The X-ray absorption spectroscopy (Cu K- and Fe K-XANES and EXAFS) of the Cu,Fe sulfide part in comparison with chalcopyrite [69] concurs with X-ray and electron diffraction, including (Fe, Cu)-S bonds of 0.230 nm and 0.271 nm (the latter is absent in chalcopyrite), but could not distinguish Fe and Cu positions. Evans and Allmann [42] believed that the brucite layers are positively charged, as this is typical for Mg minerals, especially with Al^3+^ replacing Mg^2+^, and the sulfide layers bear a negative charge.

Although valleriite is not widespread, the ores with up to 20% of valleriite enriched in platinum group metals amount about 8% of total resources of the Noril’sk ore deposit in Siberia, Russia [70,71,72]; these are not in commercial exploitation due to the lack of processing technologies.

### 2.3. Tochilinite

**Figure 5:**
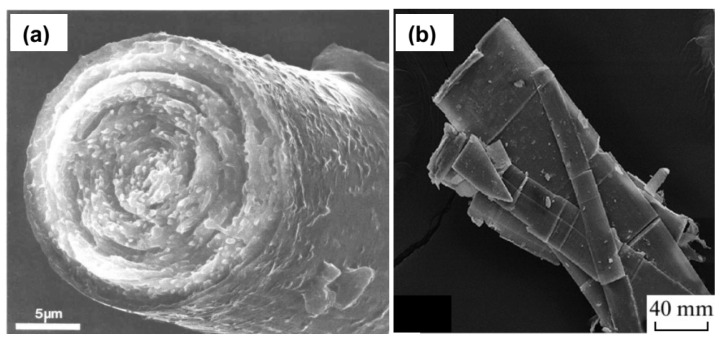
SEM images of (**a**) tochilinite fibers from Cornwall, Pennsylvania [78] (permission from the Mineralogical Society of America), and (**b**) ferrotochilinite from Noril’sk, Russia [80] (the scale length is probably 40 μm but not 40 mm) (permission from Springer Nature).

**Figure 6:**
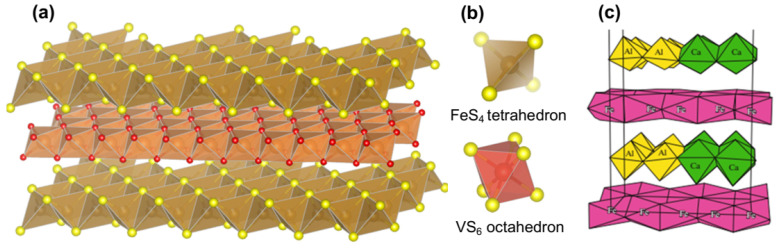
(**a**) Structure of tochilinite, (**b**) coordination units FeS_4_ in tochilinite and VS_6_ in yushkinite, and (**c**) structure of vyalsovite [85], permission from Springer Publishing Company.

Tochilinite was initially described as fibrous iron sulfide containing magnesium and water [43,73,74,75] before its valleriite-type layered structure was elucidated by Organova et al. [44,45,76]. The mineral found in many terrestrial locations has no industrial importance but is interesting because of its abundance in meteorites, particularly CM carbonaceous chondrites, and cosmic dust [77,78,79]. Tochilinite entities often have a tubular shape (Figure 5a). The composition of tochilinites can be described as 2Fe_1−x_S·*n*(Mg,Al,Fe)(OH)_2_, where 0.08 ≤ *x* ≤ 0.28 and 1.58 ≤ *n* ≤ 1.75 [44,45,76,80]. The mackinawite-like sulfide layers consist of Fe atoms located in a plane and coordinated with four sulfide anions; the distorted tetrahedra have a different orientation than in valleriite (compare Figure 3, Figure 4 and Figure 6). Iron occurs as singlet Fe^2+^ in the sulfide layers of natural tochilinites, probably forming under strongly reducing conditions [77,78,79,80], while in synthetic samples, as well as in hydroxide layers, both Fe^2+^/Fe^3+^ centers can be presented, as discussed in the next sections.

There are several known varieties of natural tochilinites, which differ in the ratio *n* of sulfide and hydroxide components and their mutual lattice arrangement, both commensurate and incommensurate [65]. Organova and co-workers [44,45,65] specified an isometric variety with the composition 6Fe_0.9_S·5(Mg_0.71_Fe_0.29_)(OH)_2_ crystallized in the space group C1. In acicular variety 6Fe_0.8_S·5[Mg_0.7_Fe_0.3_(OH)_2_], the sulfide sublattice has the space group P1, *a* = 0.834 nm, *b* = 0.854 nm, *c* = 1.074 nm, α = 87°20′, β = 94°30′, γ = 92°, and a brucite-based sublattice with the space group C1. A mineral with Fe completely replacing Mg in the hydroxide part (ferrotochilinite) was reported [80] to be monoclinic (the space group is C2/m, Cm or C2). The X-ray diffraction reflections with large *d* values, i.e., *d*_001_ = 1.083 nm and *d*_002_ = 0.539 nm, are signatures of the layered structures of tochilinite.

### 2.4. Other Minerals of Valleriite Group

A few valleriite-like minerals with different chemical compositions are known too. In 1973, haapalaite having the composition [Fe_1.26_Ni_0.74_S_2_]·1.61[Fe_0.16_Mg_0.84_(OH)_2_] and the hexagonal lattice constants of *a* = 0.364 nm and *c* = 3.402 nm was found in Outokumpu serpentines [81]. The authors suggested that Ni completely substitutes Cu in a valleriite-type structure; chemical bonding in the sulfide layers and other characteristics of haapalaite remain poorly studied.

Yushkinite [(Mg_0.6_Al_0.3_V_0.1_)(OH)_2_][V_0.875_S_2_] was discovered in 1984 [82] and has been re-examined recently [83]. The hydroxide and sulfide layers are revealed to be commensurate in the basal plane of the trigonal elementary cell (*a* = 0.325 nm, *c* = 1.14 nm, the space group *P*$\bar{3}$*m*1). Interestingly, the sulfide layer of yushkinite is composed of VS_6_ octahedra but not tetrahedra.

Vyalsovite FeS·CaAl (OH)_5_ is a very rare hydrosulfide mineral found in 1989 in the Noril’sk ore provenance [84]. Soboleva et al. [85] concluded that its structure is formed by commensurate iron sulfide and Ca,Al hydroxide layers (elementary monoclinic cells with *a* = 0.5205, *b* = 2.140, *c* = 1.440 nm, β = 95°, space group Cm). The structural model suggests that the sulfide layers consist of FeS_6_ octahedra, in contrast to tochilinite, and Al and Ca sites in the hydroxide layers are ordered (Figure 6c).

Pekov et al. [86] reported in 2014 on three new minerals of the valleriite group, which contain molybdenum and niobium in the sulfide layers. Nb is a major element in the sulfide blocks of ekplexite (after a Greek word for “surprise”) (Nb,Mo)S_2_·(Mg_1−x_Al_x_)(OH)_2+x_, while Mo prevails in kaskasite (Mo,Nb)S_2_·(Mg_1−x_Al_x_)(OH)_2+x_ and manganokaskasite (Mo,Nb)S_2_·(Mn_1−x_Al_x_)(OH)_2+x_, with a share of Mo replaced with W. In the latter mineral, Mg^2+^ is completely substituted with Mn^2+^, and it is unclear whether solid solutions exist in the system. All three structures are incommensurate with trigonal sulfide (space group *P*$\bar{3}$*m*1, *a* = *b* = 0.322–0.323 nm) and hydroxide (P3m1 or P321, *a* = 0.3066–0.3118 nm) sublattices, *c* = 1.144–1.161 nm; the replacement of Mg for Mn increases the hydroxide cell constants due to larger Mn^2+^ cation. Pekov and co-authors also summarized [80,86] the parameters of crystalline structures and X-ray powder diffraction data for all known vallerite-type minerals.

Chemical bonding in the natural layered materials remains insufficiently understood because of their complex and variable composition, contamination caused by fine intergrowth with different minerals, and the lack of big enough crystals, making it difficult to use spectroscopic techniques and to measure the physical and chemical properties. To overcome these problems, efforts to synthesize valleriite-type materials have been undertaken. Most of the synthetic work, however, has been aimed at understanding the mineral formation processes, and researchers have been interested in specifying the reaction products but not just preparing pure target substances.

## 3. Synthesis of Heterolayered Materials

### 3.1. Syntheses of Valleriites

The first laboratory preparation of valleriite was performed soon after the mineral crystalline structure was determined [42]. Iiishi et al. [87] obtained valleriite from either natural chalcopyrite or mixtures of Fe and Cu compounds with S and Mg and Al oxides in sealed gold or silver capsules at temperatures between 400 °C and 700 °C (water vapor pressure of 1000 Bar) over 5–20 days. Valleriite formed below 600 °C with chalcopyrite, brucite, pyrrhotite, covellite as by-products; the composition of valleriites was not firmly determined owing to its minor quantities. Later, Takeno and Moh [88] synthesized valleriite with Se fully or partially replacing S at 450 °C by using the same hydrothermal set-up. Preliminary prepared selenian chalcopyrite or cubanite or natural cubanite CuFe_2_S_3_ and Mg and Al hydroxides were starting reagents. Valleriite and selenian valleriite formed as submicrometer particles together with metal sulfides (selenides) and korshunskite Mg_2_Cl(OH)_3_·4H_2_O.

**Figure 7:**
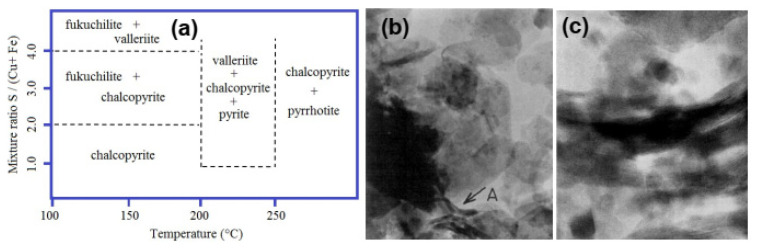
(**a**) Phase diagram showing hydrothermal formation of valleriite and other compounds, and (**b**,**c**) TEM micrographs of valleriite particles: (**c**) shows enlarged area A. Lines bounding the different phase regions were not determined accurately and are given as guides only [89]. Permission from Elsevier.

Hughes, Kakos et al. [89] conducted a successful hydrothermal synthesis of valleriite starting from co-precipitated Fe/Cu sulfides and Mg/Al hydroxide gels in the temperature range from 110 °C to 300 °C. The authors reported that valleriite formed if both hydrogen pressure (typically 10 Bar) and a large excess of sulfur (as ammonia sulfide) relative to CuFeS_2_ composition were applied (Figure 7). Almost pure valleriite phase was produced as platy crystals up to 200 nm in the lateral size and 15 nm thick using the atomic ratios of precursors Cu 2, Fe 2, S 14.6, Mg 2.08, Al 0.98 and initial pH of the mixture of 8.8 after heating at 110 °C for 25 days. X-ray diffraction, electron microscopy and X-ray photoelectron spectroscopy confirmed the structure consisting of alternating brucite-like (Mg,Al) hydroxide layers and Cu-Fe-S sulfide layers. EDS analysis of the valleriite prepared with different reagent ratios found the composition range of 1.67[Mg_0.70_Al_0.30_(OH)_2_]·[CuFeS_2_] to 1.35[Mg_0.70_Al_0.30_(OH)_2_]·[CuFeS_2_].

Chistyakova and co-workers [90,91,92] prepared valleriite from aqueous solutions with different proportions of Fe(II), Cu(II) and Mg sulfates mixed with solutions of Na_2_S taken in stoichiometric amounts. Valleriite emerged at 150 °C and 180 °C but not at 250 °C. Higher yields of valleriites were obtained for the Cu:Fe:Mg proportions of 1:2:2, 1:4:2, 3:1:4, but in all the experiments, valleriite was mixed with chalcopyrite and Fe (hydr)oxides by-products, and its content was lower than 50%, as determined by Mössbauer spectroscopy.

**Figure 8:**
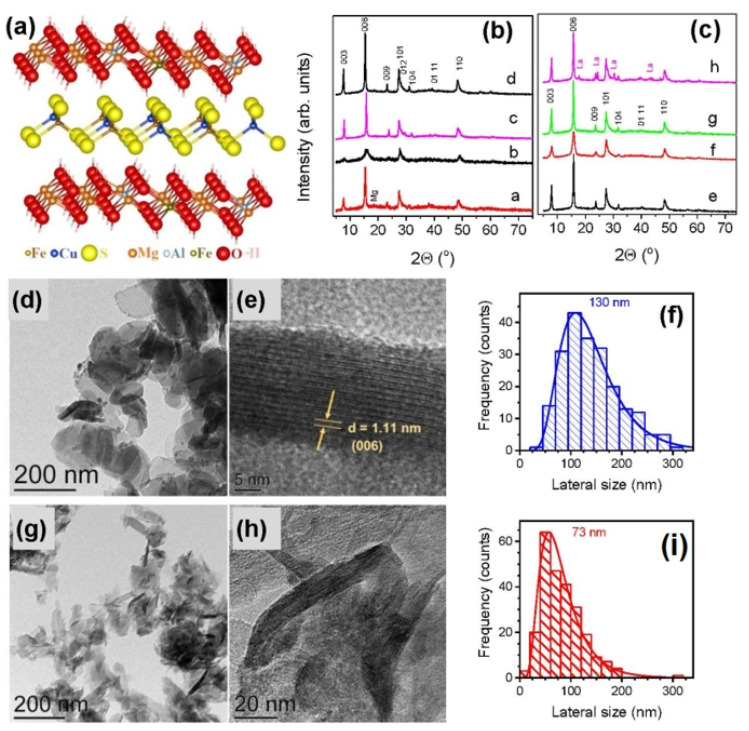
(**a**) Atomic structure of the Cu-Fe sulfide and (Mg,Fe,Al)(OH)_2_ sheets in valleriite (slightly tilted for better view), (**b**) X-ray diffraction patterns of valleriite samples synthesized a—without Al, b—with Al and c, d—various initial ratios of Fe, Cu, Mg precursors. XRD data in panel (**c**) illustrate the effect of addition of e, f—Cr, g—Co and h—La. Reflections of Mg and La hydroxides are marked as Mg (a) and La (h). TEM images (**d**,**e**,**g,h**) and particle size distributions (**f**,**i**) are given for the samples a and b, respectively [93]. Permission from RSC.

Recently, Mikhlin, Borisov et al. [93] developed a simple protocol for one-pot hydrothermal manufacturing of pure valleriite (Figure 8b,c) by mixing sulfates of iron, copper, magnesium, aluminum and, if necessary, other metals with sodium sulfide solutions and aqueous ammonia, precipitating Mg and Al hydroxides and supporting a slightly reducing atmosphere upon heating. The reaction was conducted at 160 °C during 8–100 h; the initial and final pHs were about 9 and ~12, respectively. The synthetic procedure typically involved a large excess of sulfide anions, with the initial atomic ratios S/(Fe + Cu) = 2–5, similar to the study in [89], but not hydrogen. The S excess could be almost excluded, but this resulted in minor impurity phases (mainly brucite) in some formulations. Valleriite was obtained as nanoflakes of 100–200 nm in the lateral size and 10–20 nm thickness (Figure 8d–f), which easily formed colloidal solutions during washing. Both solid precipitates and colloid particles were characterized using a set of techniques. It was shown that Mg in hydroxide layers of valleriite can be partially replaced with Al and Li, which decreases and increases, respectively, the content of Fe in the layers. The sulfide part can be doped with Co, Ni, Cr and some other metals, which partially entered hydroxide layers too.

### 3.2. Tochilinite-Type Materials

**Figure 9:**
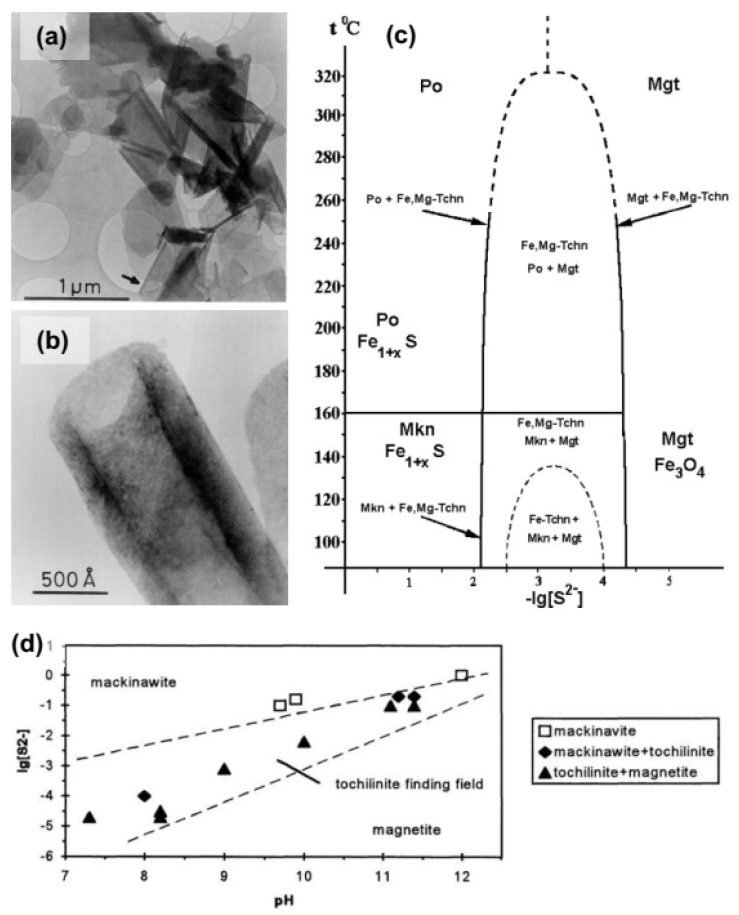
(**a,b**) TEM images of plate-like and tubular tochilinite particles prepared under hydrogen pressure and phase diagrams for the formation of (**c**) Mg,Fe-tochilinite [94] (permission from Elsevier) and (**d**) ferrotochilinite [95] during the interaction of H_2_S with metal hydroxides.

More efforts were made to synthesize tochilinite than valleriite because the former is interesting also for cosmochemistry. Kakos et al. [94] have utilized mixtures of FeS suspension prepared with iron (II) perchlorate and ammonium sulfide, and Mg/Al gels precipitated with ammonia. The mixtures were heated at 200 °C for two days under high hydrogen pressure (2 MPa). The products were tube-like and plate-like tochilinites (Figure 9a,b) with an average composition of 2Fe_1−x_S·1.7(Mg_0.7_Al_0.3_)(OH)_2_ that slightly varied in the particles of various morphology. Electron diffraction allowed one to distinguish four incommensurate structural modifications of the tochilinites: one, for plate-like crystals, was isostructural with valleriite, and three others were similar to plate-like and tubular natural tochilinites. The hexagonal hydroxide (“hydrotalcite”) layer was suggested to be charged negatively and the sulfide ones to be positive, contrary to valleriite [42,89]. Noteworthy, the authors neglected iron in the hydroxide layers both of valleriite [89] and tochilinite [94].

Kozerenko and co-workers [95] reported on a laboratory synthesis of ferrotochilinite as a product of interaction of Fe(II) hydroxides with H_2_S at 80 °C. Ferrotochilinite was obtained as a poor crystalline material mixed with magnetite and mackinawite (Figure 9d). To increase crystallinity, the reaction time was prolonged to more than 60 days and pH was enhanced. In an improved technique [96], a water suspension of ferrous hydroxide with admixture of metallic Mg (Fe:Mg = 4:1) was purged with gas H_2_S for precipitation of iron sulfides, and then tochilinite was synthesized at 120–140 °C during 10 to 45 days. The data were summarized and clarified in Ref. [97]. The formula of ferrotochilinite and more stable ferromagnesium tochilinite were specified as 2FeS·1.51(Fe^2+^, Fe^3+^)(OH)_2_ and 2FeS·1.54(Fe_0.7_Mg_0.3_)(OH)_2_. At pH 11.5–12.5, an additional phase containing Na and having tetrahedral lattice of hydroxide constituent was found; it decomposed above 100 °C with formation of mackinawite.

Chistyakova, Gubaidulina et al. [90,91] synthesized tochilinites, preferentially for Mössbauer spectroscopy studies, based on the method [96,97], i.e., via the interaction of Fe(II)-hydroxide with H_2_S at pH 11–11.5 at temperatures of 160 and 180 °C, varying the initial quantities of Mg. The yield of tochilinite increased with increasing content of Mg in the reaction mixture but stayed well below 50% as considerable impurities of magnetite, troilite and pyrite were produced.

Peng et al. [98,99,100] produced tochilinite and tochilinite–serpentine-intergrowth phases simulating the components of meteorites. The alloyed metal particles of Fe, Mg and Al [98], or more complex mixtures [100], were preliminary prepared. The precursors were then reacted with aqueous ammonium sulfide or elemental sulfur at pH 13–14 below 200 °C. Tochilinite was obtained as nanoflakes and tubes with the content in the solid products less than 40%. “Sodium-tochilinite”, which has a tochilinite structure with Na hydroxide layers containing Fe, was synthesized from Fe metallic powder and sodium sulfide at 98–150 °C during 30–15 days [99]. The authors identified four varieties of Na-tochilinite, which were essentially disordered and not quite stable. It was reported that the sodium hydroxide layers can be completely removed with water or weak acids, leaving mackinawite-like Fe_1−x_S phase. Less stable varieties of Na-tochilinite entered reactions with a number of such substances as NH_3_, N_2_H_4_, N_2_, 2,2′-bipyridine and 1,10-phenanthroline, forming several types of intercalates, mainly replacing sodium hydroxide layers.

Vacher et al. [79] have obtained tochilinite along with cronstedtite and other minerals under conditions modeling their formation in CM chondrite meteorites, starting from metallic iron and mineral assemblages. Iron-rich tochilinite surrounded the grains of metallic Fe reacted in S-bearing solutions at 80–120 °C; content of Mg in the hydroxide layers increased with temperature.

**Figure 10:**
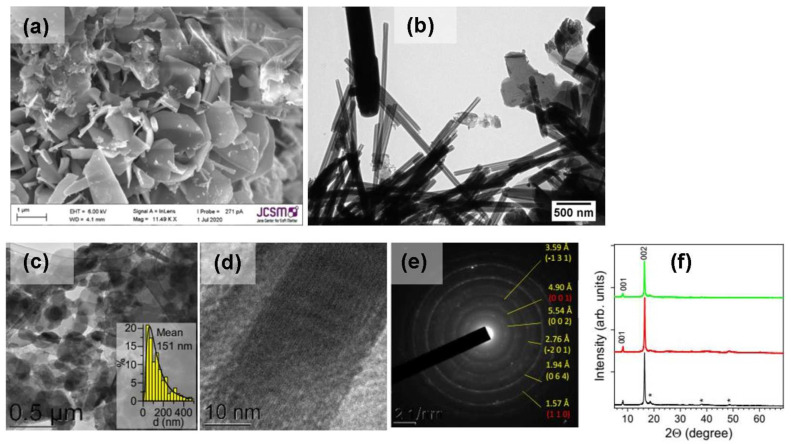
Upper panels: (**a**) SEM and (**b**) TEM images of Fe_0.76_S⋅0.86 [Fe^2+^_0.01_Fe^3+^_0.56_Mg^2+^_0.43_(OH)_2.01_] prepared using elemental Fe and S [101]. Lower panels: (**c**,**d**) typical TEM images, (**e**) electron diffractogram and (**f**) X-ray diffraction patterns of tochilinites prepared using atomic proportions of reagents Fe 2, Mg 1.5, S 15 (black), Fe 2, Mg 1.5, S 15, Al 0.5 (red), and Fe 2, Mg 1.5, S 15, Li 0.5 (green); asterisks designate reflections of brucite [103]. Reproduced with permission from RSC.

Tochilinites with various hydroxide layers have recently been synthesized by Bolney et al. [101], applying the hydrothermal method developed for mackinawite [102]. For the preparation of magnesium tochilinite with the composition Fe_0.76_S·0.86[Fe^2+^_0.01_Fe^3+^_0.56_Mg^2+^_0.43_(OH)_2.01_], a water suspension of FeS nanoparticles obtained at 80 °C using elemental S and Fe at the atomic ratio of 1 [102] was mixed with a suspension of powdered magnesium and FeO(OH) and heated at 160 °C for 3 days. Aluminum tochilinite Fe_0.89_S·0.85[Fe^2+^_0.55_Fe^3+^_0.11_Al^3+^_0.33_(OH)_1.84_(O)_0.16_] was prepared from a finely ground mixture of elemental Fe, S and Al in water at 130 °C over 3 days. Ferrotochilinite Fe_0.71_S·0.79[Fe^2+^_0.25_Fe^3+^_0.73_Mg^2+^_0.01_Al^3+^_0.01_(OH)_1.98_(O)_0.02_] was synthesized similarly from iron and sulfur taken in a mass ratio 3:1. In all the cases, excess of Fe was used to support hydrogen pressure (along with metallic Mg and Al), and to suppress the formation of pyrrhotite and pyrite. By-products (magnetite) and unreacted Fe metal were separated by a magnet or/and washed out (brucite and others). The tochilinites were submicrometer platelets of a few nm in thickness with admixtures of nanotubes (Figure 10, upper photos). The tochilinite samples were monophase after the purification; the XRD patterns were dominated by two strong reflections, the d-values of which at approximately 11 Å and 5.5 Å were assigned to the (001) and (002) peaks, as previously reported for natural tochilinite. All other diffraction peaks had little intensity, which might be caused by preferred orientation or turbostratic stacking of the layers [101]. The abovementioned distribution and oxidation state of Fe were derived from Mössbauer spectra, which suggest that singlet Fe^2+^ cations occur in the sulfide layers akin to natural minerals, and all the hydroxide layers comprise high numbers of Fe^2+^/Fe^3+^ centers.

Tochilinites with magnesium hydroxide layers, including doped with Al and Li, were prepared [103] using aqueous solutions of metal sulfates, sodium sulfide and ammonia in a simple hydrothermal method similar to that developed for valleriite [93]. The iron sulfide and Mg, Al, Li hydroxides were loaded in an autoclave with Teflon liner and heated at slow rotation (8 rpm), typically at 160 °C for ~40 h. The reaction media were purged with Ar before sealing the reactor, but no reductants (elemental metals, hydrogen, etc.) were employed, in contrast to other works. The atomic ratios of S and Fe precursors varied from 1 to 10, and usually, a large excess of sodium sulfide was used (and removed during decantation washing of the solid products), allowing one to obtain pure tochilinite with no admixtures of iron sulfide or (hydr)oxide phases. The samples prepared with nearly stoichiometric initial amounts of sulfide contained minor impurities of brucite. Tochilinite formed as nanoflakes of 100–200 nm in the lateral dimensions and 10–15 nm thickness (no tubular particles were observed), which were insignificantly affected by precursor ratios, temperature and reaction time. It was found that Al and Li entered Mg hydroxide layers proportionally to their contents in the reaction mixtures, decreasing and increasing, respectively, the concentrations of Fe in the hydroxide part of tochilinite; characteristic examples can be described by the formula Fe_0.77_S·1.14[(MgFe_0.2_)(OH)_2.22_], Fe_0.85_S·0.87[(MgAl_0.25_Fe_0.17_)(OH)_2.75_] and Fe_0.7_S·0.81[(MgLi_0.3_Fe_0.5_)(OH)_1.5_O_0.05_]. The synthetic tochilinites were examined using, in addition to TEM, EDS, XRD and electron diffraction, a number of experimental techniques described below.

### 3.3. Layered Superconductors of FeSe·(Li,Fe)(OH) Group

Superconductivity below T_C_ = 8.5 K discovered in tetragonal iron selenide β-FeSe by Hsu et al. [60] in 2008 attracted new interest to layered iron chalcogenide materials. The critical temperature T_C_ depends on the substitution of Se with Te or S, stoichiometry of the iron chalcogenide and ordering the Fe vacancy system, the interlayer distance and other factors. Moreover, single-layer FeSe films grown on SrTiO_3_ (001) surfaces exhibited the temperature T_C_ as high as 109 K [104]. A number of superconductors with metal cations or molecules intercalated between the FeSe sheets weakly bonded by vdW forces, including A_x_Fe_2−y_Se_2_, where A stands for K, Rb, Cs, were synthesized [105,106].

**Figure 11:**
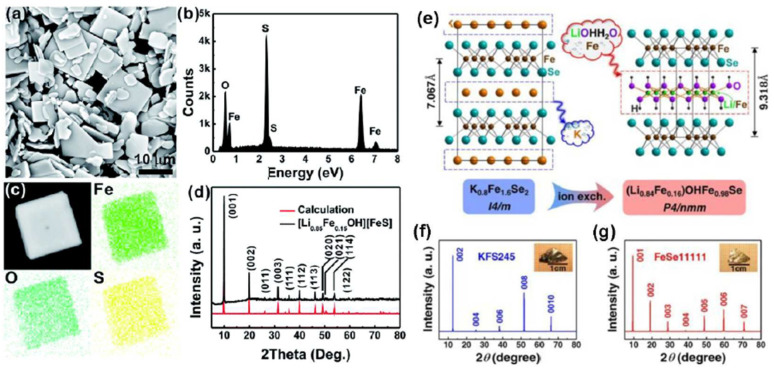
Left panels: (**a**) FESEM of [Li_0.85_Fe_0.15_OH][FeS] single crystals. (**b**) EDX results of the single crystals. (**c**) Elemental distribution of the Fe, O and S elements in the [Li_0.85_Fe_0.15_OH][FeS] single crystal. (**d**) X-ray diffraction of [Li_0.85_Fe0.15OH][FeS] [111] (permission from RSC). Right panels: (**e**) a schematic illustration of structural changes in the process of the hydrothermal ionic exchange reaction with the starting materials of big matrix crystals of K_0.8_Fe_1.6_Se_2_, LiOH·H_2_O, Fe and CH_4_N_2_Se. (**f**,**g**) The XRD patterns of (00l) type for the K_0.8_Fe_1.6_Se_2_ and the (Li_0.84_Fe_0.16_)OH·Fe_0.98_Se crystals, respectively, demonstrating their crystal orientations along (001) planes. The insets show the corresponding photographs of the crystals. Reproduced from [115] with permissions of APS.

Lu and coworkers [107] reported in 2015 on a hydrothermal synthesis of polycrystalline (Li_0.8_Fe_0.2_)OHFeSe with T*c*~40 K, constructed from alternating layers of the distorted FeSe tetrahedra and Li-based hydroxide. Interestingly, the authors initially missed the existence of (Li,Fe)OH sheets and considered the spacer layer as LiFeO_2_ [108]. The structure strongly resembling tochilinite was obtained using metallic Fe powder, selenourea, in large excess in relation to FeSe, and a big amount of LiOH, that is, under essentially reducing and strongly alkaline conditions, at 160 °C over 3–10 days. In contrast to FeSe intercalated with alkaline metals, ammonia and their complexes, these materials are principally stable in air. With some variations, this method was then utilized by many researchers for the preparation of [(Li_1−x_Fe)OH][(Fe_1−y_Li_y_)Se], [Li_0.85_Fe_0.15_OH][FeS] and other candidate superconductors with slightly varying compositions of hydroxide and chalcogenide layers, as powders with the crystallites on the order of several decades of μm [109,110,111,112,113,114] (Figure 11).

Large, 1–2 cm, single crystals of FeSe (Li_1−x_Fe_x_)OH and similar compounds were synthesized using the “ion exchange” procedure with A_x_Fe_2−y_Se_2_ (A = K, Rb, and Cs) single crystals, grown by self-flux, Bridgman, or other methods, as precursors [115,116]. In a typical procedure, the precursor crystals and the reaction mixture of selenourea, Fe powder (now with excess of Fe) and LiOH were loaded into a Teflon-lined autoclave and heated to 120–200 °C for several days. Using a similar protocol, Guo et al. [117] manufactured single crystals of layered Fe_1−x_S (NaOH); the authors did not mention Fe in the sodium hydroxide part, and admitted its sensitivity toward ambient air.

The above studies of layered superconductors (more references and discussion can be found, for example, in [118] and the literature cited above) are very focused on structural, magnetic and other phenomena related to superconductivity, taking into consideration only few chemical compositions and almost ignoring additional characteristics and applications of this class of materials. Certainly, the advances in the syntheses must be employed for preparation of other heterolayered substances.

### 3.4. Summary on the Synthesis

Brief results on the synthesis of valleriite-type materials are collected in Table 1. The total number of such studies is not large, except for the preparation of the superconducting materials (only few examples are shown). Mostly, the target of the research was to shed light onto mineral formation mechanisms, and the layered hybrids were mixed with other solid products, sometimes unintentionally, because of imperfect synthetic protocols. Pure valleriite phases were successfully fabricated using an excess of sulfur or/and selenium [89,93]. The hydrogen pressure and highly reducing conditions [89] are not mandatory for valleriites, but they likely influence some structural peculiarities and properties, which needs further investigation. Tochilinites were produced under such conditions too; the hydrogen atmosphere is essential for obtaining low-spin Fe^2+^ centers in the chalcogenide layers. The syntheses of FeSe(Li,Fe)OH-type superconductors [107,109] were effectively performed using metallic iron and selenourea (thiourea for FeS layers), which react slowly and probably impede nucleation of iron chalcogenides. This causes the growth of rather big particles, up to several decades of micrometers (often single crystalline), instead of nanoflakes and nanotubes for chalcogenide anions as precursors. The synthesis of large single crystals is possible via the multistep “ion exchange” procedure [115,116], which has not been applied for valleriites yet.

## 4. Spectroscopic Characterization and Properties

### 4.1. Mössbauer, XPS, X-ray Absorption Spectra of Valleriite

Whereas the crystalline structures of layered materials are rather well understood [42,63,64], a number of major questions regarding the chemical bonding and state of elements, particularly Fe, and their physical and chemical properties are still unresolved. The spectroscopic studies of natural minerals and synthetic samples are rare and often the results are not unambiguous.

^57^Fe Mössbauer spectroscopy, X-ray photoelectron spectroscopy or their combination have been applied to study the oxidation and spin state of iron atoms in sulfide (chalcogenide) and hydroxide blocks. Hughes et al. [89] concluded from XPS spectra that the submicrometer particles of Al-containing valleriite synthesized under hydrogen pressure contain Cu^+^ and Fe^3+^ centers. Waanders and Pollak [119] published room-temperature Mössbauer spectra acquired from two natural valleriite crystals replacing chalcopyrite and associated with magnetite, and having the compositions [Cu_0.94_Fe_1.06_S_2_]·1.4[Mg_0.8_Fe_0.20_(OH)_2_] and [Cu_1.02_Fe_0.98_S_2_]·1.72[Mg_0.69_Fe_0.05_Al_0.26_(OH)_2_], respectively. The spectra consist of central signals fitted with four doublets, one of which (isomer shifts δ of 0.28 or 0.22 mm/s and quadrupole splitting Δ of ~0.3 mm/s) was assigned to Fe^3+^ centers in hydroxide layers (22% and 10% of total iron in these valleriites, respectively). The others were attributed to two Fe^3+^ centers (δ = 0.25–0.4 mm/s and the quadrupole splitting Δ = 0.74–0.89 mm/s) and Fe^2+^ centers with δ = 0.4–0.42 mm/s and Δ = 1.12 and 1.32 mm/s (about 32% of total iron) orderly arranged in tetrahedral positions in sulfide layers. Chistyakova et al. [90] reported Mössbauer spectra of several natural and synthetic valleriites notably contaminated, but with impurity phases, particularly chalcopyrite. The authors suggested that doublets δ = 0.36 ± 0.03 mm/s and ε = Δ/2 = 0.30 mm/s, and δ = 0.39 ÷ 0.54 mm/s and ε = 0.46 ÷ 0.56 mm/s are due to Fe in sulfide layers of valleriite, while quadrupole doublets with parameters δ ≈ 1.1 mm/s and ε ≈ 1.2 mm/s could correspond to iron positions in the hydroxide layers [92].

The interpretation of the above spectra of the samples containing several Fe species both in valleriite and foreign phases is difficult and contradictive since it is based at the room-temperature Mössbauer spectra only. In Ref. [69], Mössbauer spectra were acquired at 293 K, 78 K and 4.2 K and compared with the results of X-ray absorption spectroscopy (particularly, Fe-K- and Cu K-edge XANES and EXAFS) and XPS [69,120] from two natural valleriites intergrown with pyrrhotite Fe_1−x_S and magnetite, as well as with chalcopyrite. The joint analysis of the spectra suggested that Cu^+^ and Fe^3+^ centers occur in the sulfide part of valleriites (hyperfine Mössbauer parameters δ ≈ 0.4 mm/s and Δ ≈ 0.6 mm/s at room temperature), and Fe^3+^ cations predominate in the hydroxide part. Room-temperature Mössbauer and XPS spectra [121] for valleriite associated mainly with serpentines generally agreed with this interpretation. Upon cooling down to 4.2 K [69], the central paramagnetic doublets almost disappeared, giving rise to a series of hyperfine Zeeman sextets with internal hyperfine magnetic fields *H* of *~*270, 320 and 490 kOe emerging both in sulfide and hydroxide layers of valleriites; no transitions into the antiferromagnetic state typical for Cu-Fe sulfides were observed.

**Figure 12:**
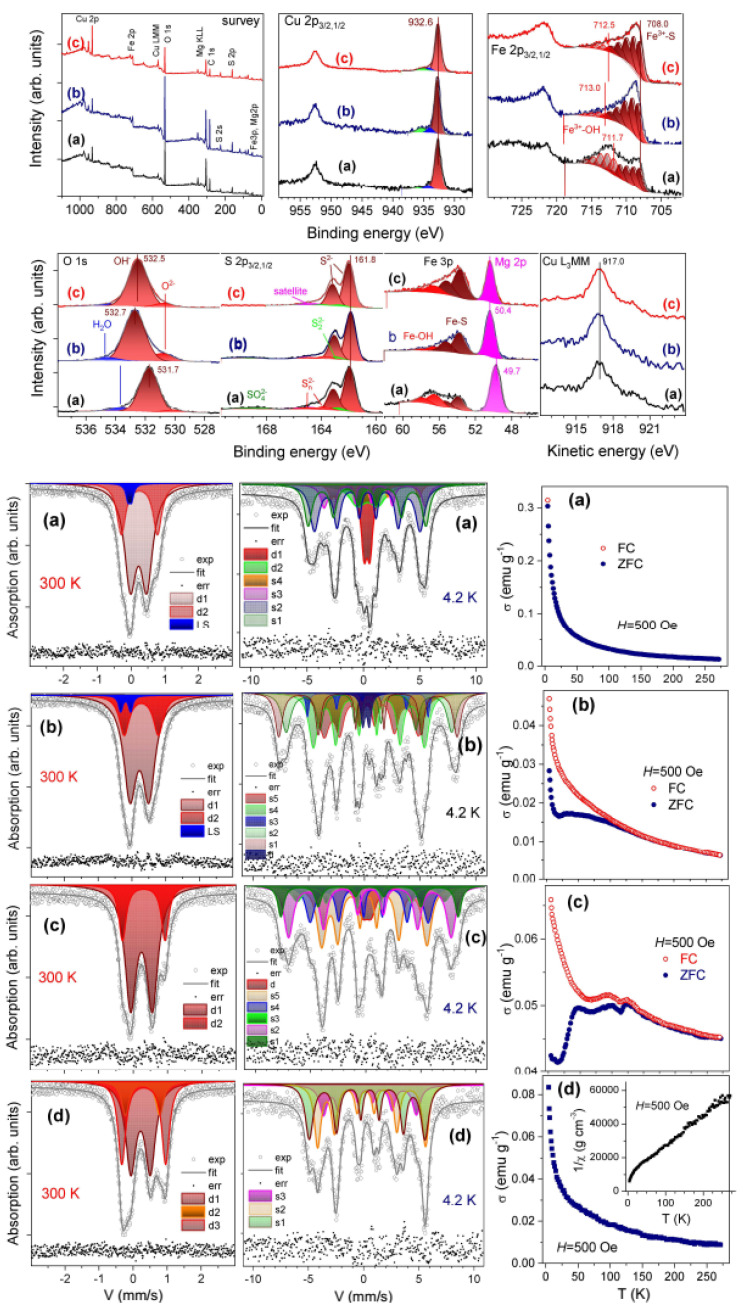
**Upper panels:** X-ray photoelectron spectra, **lower panels**: Mössbauer spectra, temperature (FC and ZFC) and field dependences (hysteresis loops at 4.2 K) of magnetization and reciprocal susceptibility 1/χ of valleriite. The samples were synthesized with different proportions of Fe and Cu precursors without Al (a) and with Al (b), (c), and with Cr (d) [93]. Permission from RSC.

Further insight was gained by exploring synthetic valleriites free of foreign phases (Figure 12) [93]. It was derived from the photoelectron Fe 2p (fitted using multiplet line sets) and Fe 3p spectra that Fe^3+^ cations predominate both in Cu-Fe sulfide and Mg-based hydroxide layers. The quantity of *“*hydroxide” Fe can be diminished by adding Al (spectrum a vs. spectra b and c) or increased by adding Li, which enter the hydroxide module during the hydrothermal synthesis, in the range from 10% to 45% of total iron; this also influenced the share of O^2−^ anions (compare O 1s spectra a and b). In addition, XPS found a higher content of sulfur (as monosulfide and minor polysulfide) than EDS analysis, suggesting that some excessive sulfide anions used in the synthesis were adsorbed on the nanoflake surfaces.

These findings were used to better understand Mössbauer spectra. It was confirmed that the room-temperature doublets with δ = 0.3–0.4 mm/s and Δ = 0.5–0.6 mm/s, and δ~0.3–0.4 mm/s and Δ of 1–1.2 mm/s should be assigned to Fe^3+^ centers in the tetrahedral coordination with S and octahedral coordination with OH^−^ groups, respectively. Several sextets with distinct hyperfine fields at low temperatures are indicative of distinct Fe sites both in sulfide and hydroxide sheets. The sextets with *H* of 300–330 kOe were attributed to sulfide layers, and those with *H*~270 kOe were ascribed to Fe-OH centers. The assignment of smaller sextets with *H*~500 kOe (spectra b, c) to Fe-S centers was probably erroneous, since those are likely due to Fe^3+^-(OH^−^)_x_O^2−^_y_ centers involving some O^2−^ anions seen in O 1s spectra, as it was later established for tochilinite [103]. Magnetic measurements revealed generally paramagnetic behavior that becomes more complex if the content of Fe in the hydroxide layers decreases (Figure 12, samples b and c).

### 4.2. Mössbauer and XPS Investigation of Tochilinite

There is agreement in the literature that Fe cations in sulfide layers of natural tochilinite [77,78,79,80,90,91,101,122] are in the low-spin Fe^2+^ state, similar to mackinawite [55,56,57,58,59,102], exhibiting a narrow Mössbauer doublet with isomer shifts δ in the range from 0.4 to 0.45 mm/s and a small quadrupole splitting Δ (0.18 mm/s to 0.24 mm/s). The maxima are retained at cryogenic temperatures. The hyperfine parameters for Fe^2+^ and Fe^3+^ octahedral centers in hydroxide layers were determined to be δ of ~1.2 mm/s and Δ = 2.2–2.3 mm/s, and δ of ~0.3 mm/s and Δ = 0.7–1 mm/s, respectively. These findings allowed for the use of Mössbauer spectroscopy for the analysis of concentrations of iron in tochilinites. Many authors have taken for granted that iron occurs in hydroxide layers as Fe^2+^, but this seems to be incorrect. Particularly, Bolney et al. [101] found with EDS analysis and Mössbauer spectroscopy that in tochilinites prepared under essentially reducing conditions, Fe^3+^ cations predominated in the Mg-based hydroxide layers; preferentially, Fe^2+^ centers were found in Al-containing layers [Fe^2+^_0.55_Fe^3+^_0.11_Al^3+^_0.33_(OH)_1.84_O_0.16_] and a (Fe^2+^_0.25_Fe^3+^_0.73_)(OH)_1.98_O_0.02_ proportion was reported for ferrotochilinite. X-ray photoelectron Fe 2p spectra, which should have a characteristic strong narrow peak from singlet Fe^2+^at 707 eV, were rarely reported. Moreover, Zhang et al. [111] published the XPS spectra of single crystals of Fe_1−x_S (NaOH), which did not show such a peak and were explained in terms of (Fe^3+^, Fe^2+^)-S centers.

Tochilinite-type materials synthesized without metallic iron [103] have Mössbauer spectra resembling those of valleriite, that is, asymmetric doublets in room-temperature spectra and at least three Zeeman sextets at 4 K. The doublets with the isomer shift δ = 0.3–0.4 mm/s and quadrupole splitting Δ = 0.4–0.6 mm/s are due to Fe^3+^-S centers, and the ones with δ ≥ 0.4 mm/s and Δ = 1 ± 0.2 mm/s originate from high-spin Fe^3+^ cations in the octahedral OH^−^ environment, whereas the quantities of Fe^2+^-OH centers (δ~1 mm/s, Δ > 2 mm/s [90,91,92]) are minor. However, the signals of Fe^2+^ cations in tetrahedral coordination to S and those of Fe^3+^-6OH centers having similar parameters are difficult to resolve. The sextets (hyperfine fields of ~290, 350 and 480 kOe) indicating magnetic ordering at low temperatures were attributed to high-spin Fe^2+^-4S together with Fe^3+^-(6OH), centers Fe^3+^-4S, and Fe^3+^(OH)_x_(O)_y_ centers, respectively, in hydroxide layers. Photoelectron Fe 2p spectra are not quite clearly fitted too due to the co-existence of comparable numbers of high-spin Fe^3+^ and Fe^2+^centers in the sulfide part. It was established, nevertheless, that the hydroxide layers preferentially contain Fe^3+^ cations, whose amount also depends on the addition of Al and Li. Combined analysis of Mössbauer, XPS and UV-vis spectra (see below) suggested that comparable quantities of high-spin Fe^2+^ and Fe^3+^ cations are presented in the sulfide layers of such tochilinites.

### 4.3. Raman, IR Spectroscopy, UV-Vis-NIR Spectroscopy

Raman spectroscopy is an important instrument for the characterization of 2D materials [123]. Several Raman spectra are available in the literature for natural valleriite-type minerals [63,124,125] and synthetic valleriites [93]. The spectra below 500 cm^−1^ originate from sulfide sheets; the strongest maxima at about 300 cm^−1^ both for tochilinite and valleriite are due to sulfur-only, almost independent on cations, vibrations of A_1g_ symmetry, while others are due Cu-S and Fe-S vibrations [93,126,127,128]. Full interpretation and application of Raman spectra for studying the chalcogenide–hydroxide structures are still required. The same is true for IR spectroscopy, mainly characterizing the hydroxide part [48,63,80,124,125], and optical reflection spectra, which are commonly used as a tool for identification of minerals.

**Figure 13:**
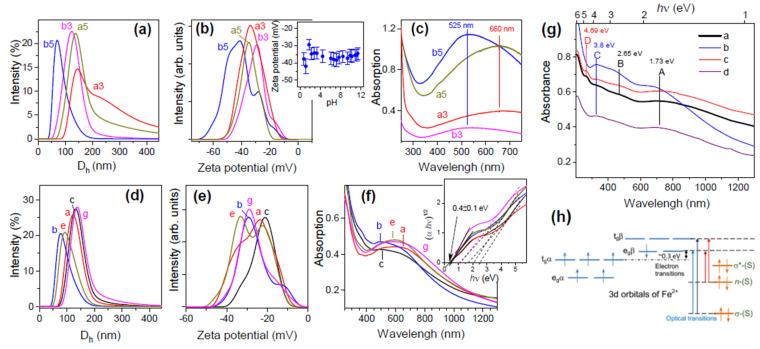
Left panels: The hydrodynamic diameters D_h_ (**a**,**d**), zeta potentials (**b**,**e**), and UV-vis-NIR absorption spectra (**c**,**f**) of valleriite hydrosols spontaneously formed during washing (upper panels (**a**–**c**) and a zeta potential—pH plot for the sample b5 in an insert) and prepared by means of sonification of corresponding residues in aqueous 2 mM SDS solution (**d**–**f**). In upper panels, the samples were synthesized (32 h) using the initial precursor ratios: a3, a5—Al 0.5, Fe 2, Cu 2, Mg 2, S 14; b3, b5—Al 0, Fe 2, Cu 2, Mg 2, S 14, with indexes 3 and 5 standing for the number of the washing stage in which the particular sol was formed. In lower panels, hydrosol sample a contained no Al; b, c—Al-containing, e—Cr-doped [93]. Right panels. (**g**): UV-vis-NIR absorption spectra of aqueous colloids of tochilinite synthesized hydrothermally using the proportions of reagents a—Fe 2, Mg 1.5, S 15, b—Fe 2, Mg 1.5, S 15, Al 0.5, c—Fe 2, Mg 1.5, S 15, Li 0.5, d—Fe 2, Mg 1.5, S 15, Al 0.5, Li 0.5; and dispersed in water. (**h**): Simplified diagram of the electron levels for Fe^2+^ cations assuming tetrahedral coordination (the levels for Fe^3+^ are not shown), S ligands, and tentative optical charge-transfer transitions in the sulfide layer of tochilinite [103]. Permissions from RSC.

The absorption UV-vis-NIR spectra [93] collected from valleriite flakes in the colloidal solutions have maxima centered at 500–700 nm and redshifted for the samples with higher contents of Fe in the hydroxide layers (Figure 13c,f). Such maxima commonly observable for metallic nanoparticles may be attributed to localized surface plasmon resonances (LSPR) [129]. This, however, looks unlikely, as the Tauc plots, as well as reflection electron energy loss spectra (REELS) [93], suggest that valleriites are semiconductors with the indirect band gap of 0.4–0.5 eV. The gap width is close to that for chalcopyrite CuFeS_2_, the nanoparticles of which exhibit similar optical spectra with the maxima attributed to the quasi-static dielectric resonance [130,131] due to the “intermediate” narrow minority-spin Fe 3d band rather than LSPR.

**Figure 14:**
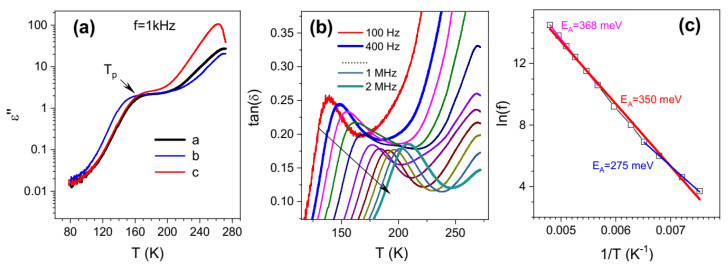
(**a**): Imaginary part of dielectric permittivity for tochilinites prepared with the atomic ratios of precursors a—Fe 2, Mg 2, S 15, b—Fe 2, Mg 2, S 15, Al 0.5, c—Fe 2, Mg 2, S 15, Li 0.5. (**b**): dielectric loss tangents at various frequencies as a function of temperature for the sample b; arrow marks increasing frequency. (**c**): peak frequency vs. reciprocal temperature (Arrhenius plot) for the sample b. Reproduced from Ref. [103] with permission from RSC.

UV-vis-NIR absorption spectra of tochilinite colloids are more complicated [103], showing broad maxima A and a set of narrower blueshifted peaks B–D. It was suggested that feature A can be interpreted as the all-dielectric Mie resonance in insulating and semiconducting nanomaterials [15,132,133]. This is associated with light scattering (both electric and magnetic components) by subwavelength particles with a high refractive index. The measurements of dielectric properties (Figure 14) [103] imply that this can be the case for tochilinites and possibly for valleriite too. On the other hand, the spectral features at shorter wavelengths likely reflect the ligand-to-metal charge transfer, involving orbitals of sulfur anions and Fe 3d states, similar to Fe-S clusters in proteins [134,135]. The clusters denoted as [1Fe-0S]^2+/3+^ with Fe^2+^ (spin number S = 2) or Fe^3+^ (S = 5/2) in tetrahedral environment of sulfur [134] better agree with the data of Mössbauer spectroscopy and XPS, thus suggesting that both Fe^2+^ and Fe^3+^ cations with insignificant alignment of neighboring electron spins exist in the tochilinite produced in not strongly reducing media [103].

### 4.4. Physical and Chemical Properties

Some basic physical properties determined experimentally or calculated are available for minerals in the literature; for example, density of 3.1–3.2 g/cm^3^ and Mohs hardness 1.0–1.5 are reported for valleriite, and the densities are ~3 g/cm^3^ and 3.47 g/cm^3^ (calculated) for tochilinite and ferrovalleriite, respectively [63]. However, critically important electronic characteristics of the materials have been very poorly studied up to now, leaving aside superconductors. Valleriite and tochilinite appear to be narrow-gap semiconductors with the indirect band gaps of the sulfide layers of about 0.5 eV and 0.3 eV, as determined from optical absorption spectra and REELS [93,103], as well as the solid-state impedance measurement for tochilinite [103].

Magnetic measurements in combination with Mössbauer spectroscopy (Figure 12) revealed that synthetic valleriite and tochilinite [93,103] are paramagnetic at room temperature and 4 K, but magnetic ordering takes place at low temperatures. The behavior is more complicated if the quantity of iron in hydroxide layers is reduced. This suggests a kind of interlayer magnetic interaction in the materials. It is worth mentioning that FeSe (Li,Fe)OH superconductors demonstrate superconductivity in chalcogenide layers and low-temperature ferromagnetism [107,109].

**Figure 15:**
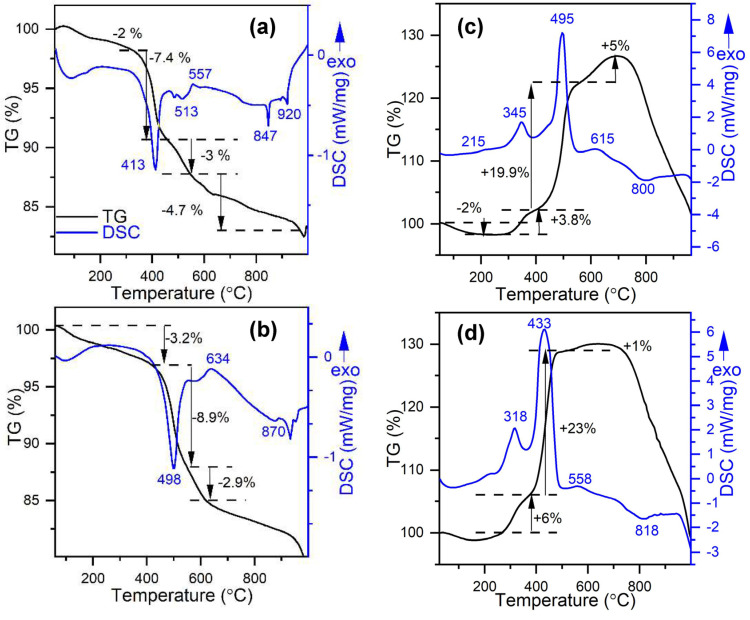
TGA and DSC profiles acquired in Ar (left panels (**a**,**b**)) and 20% O_2_ + 80% N_2_ media (right panels (**c**,**d**)) for synthetic valleriite samples prepared with no additives (**a**,**c**) or using Al (**b**,**d**) as a modifier decreasing the content of Fe in hydroxide layers. Adapted from [136]. © Distributed by Creative Commons Attribution 3.0 License.

Only selected chemical properties of valleriites have been reported. The Mg-based materials are quite stable in ambient air and under moderate heating, as it was demonstrated for natural and synthetic samples [93,101,121,136,137]. Valleriite is more resistant than tochilinite. A thorough examination of the thermal behavior of synthetic valleriite nanoflakes was conducted [136] using TG/DSC methods in inert and oxidative atmosphere up to 1000 °C (Figure 15). Dehydroxylation of the hydroxide layers was found to peak at 413 °C, and metal sulfide sheets start to degrade below 500 °C, converting to bulk Cu_5_FeS_4_, CuFeS_2_ and FeS in inert atmosphere. The exothermic reactions in oxidative media proceed with a mass increase as sulfur oxides form and react with magnesium hydroxide layers to yield MgSO_4_ (the main maximum at 495 °C). Samples doped with Al, which decreases the content of Fe in hydroxide layers, notably impeded endothermic decay of valleriite in argon; on the contrary, the rate of oxidation increased. This was explained by high thermal resistance across the stacked sheets having very different composition (masses of elements and phonon frequencies), so the large number of Fe atoms in the hydroxide sheets, comparable with that in sulfide layers, promote the phonon exchange and heat transfer between the layers, and vice versa.

Several studies have been devoted to chemical reactions of valleriite-containing materials related with mineral processing, including flotation [137], leaching and sulfidation with aqueous SO_2_ [72], deposition of precious metals from aqueous solutions [138], and bacterial leaching [139]. Some special features of the valleriite reactivity were observed, but more research is needed.

For colloidal solutions of valleriite and tochilinite synthesized using excessive sodium sulfide [93,103], zeta potentials of the particles were negative, about −30 mV, over a wide pH range. This implies a negative charge of the hydrophilic hydroxide layers, which are expected to be exposed in aqueous media, and a positive charge of sulfide layers. The effect of the precursor proportion Fe/Mg and Al or Li doping was less significant. Tochilinite manufactured with the initial ratio Fe/S close to 1 exhibits slightly negative potentials, thus underlying the role of sulfide anions in the synthesis.

## 5. New Characteristics and Potential Applications of Chalcogenide–Hydroxide 2D Materials

The layered chalcogenide–hydroxide materials comprise very different, chemically incompatible components, which often have incommensurate lattices. The compositions both of chalcogenide and hydroxide layers widely vary, and they are referred to as composites or hybrids, or heterostructures. Meanwhile, the alternating layers are ordered on a molecular level and are spatially homogeneous, despite some packing defects and imperfection within the layers. This allows one to consider valleriites as individual chemical substances rather than a combination of two compounds.

The large distances, on the order of 11 Å in comparison with 6.5 Å in MoS_2_ [11] and 5.0 Å in mackinawite FeS [54], and the absence of short-range forces between the metal sulfide sheets separated by dielectric hydroxide layers, and vice versa, suggest probable two-dimensional phenomena in the layers. On the other hand, long-range (electrostatic, magnetic) interactions controllable via characteristics of distinct layers, e.g., Fe^2+^/Fe^3+^ content in the hydroxide ones, could affect the 2D properties. The interplay of 2D and 3D factors opens ways for engineering new materials and tuning their properties, and promises novel non-trivial effects.

In contrast to vdW heterostructures, the layers are generally believed to interact via the opposite electrostatic charges. This is not directly confirmed yet, and there are contradicting opinions regarding their signs [42,44,89,93,94,103], which stem, among other things, from errors in determining the concentrations of Fe^2+^/Fe^3+^ cations in both layers, and covalent metal–sulfur and S-S bonding. Zeta potentials of synthetic valleriite [93] and tochilinite [103] are indicative of the negative charge of magnesium-based hydroxide layers, despite the replacement of Mg^2+^ with Fe^3+^ and Al^3+^ cations, maybe, owing to the formation of O^2−^ anions. It has been speculated that electroneutrality of FeSe layers and H-bonding between the layers take place in FeSe(Li,Fe)OH superconductors. However, Chen et al. [112] calculated using density functional theory that Fe atoms in (Li_0.8_Fe_0.2_)OH layers promote electron density transfer to FeSe and attraction between the chalcogenide and hydroxide sheets. One should expect that the charge signs and amplitudes, affected by the total material composition and the state of Fe, can be tuned upon the synthesis and post-synthetic treatment.

The charge difference is an apparent directing force of self-assembly of valleriites, which also can be controlled using, for instance, excessive sulfide anions or redox potential of reaction media. At present, hydrothermal synthesis with elemental metals and chalcogens or their simple compounds as precursors is the main route to powder materials composed of submicrometer flakes, tubes or micrometer plate-like crystals (Table 1). Large single crystals of few materials have been prepared via the protocol combining thermic synthesis of precursors and hydrothermal ion exchange reaction [115,116]. Further research and application of valleriites require fabrications of films, coatings and devices, in which the nanoflakes can be utilized as building blocks. Also, it would be interesting to prepare mono- and few-layer valleriites using different techniques, e.g., atomic layer deposition and chemical bath deposition. An intriguing question is whether components other than chalcogenides and hydroxides can be joined in 2D heterostructures; it is worth recalling, for example, a mineral koenenite consisting of chloride [(Na_4_(Ca,Mg)_2_Cl_12_]^4−^ and hydroxide [Mg_7_Al_4_(OH)_22_]^4+^ layers [140].

The preliminary study of magnetic properties revealed paramagnetic character of valleriites and tochilinites along with magnetic ordering at 4 K, distinguishing several Fe sites in both layers. High Fe concentrations in hydroxide sheets were found to promote paramagnetic behavior, probably due to some 3D magnetic exchange interaction. Two-dimensional materials containing different 3d transition and rare-earth metals both in hydroxide and chalcogenide layers have already been obtained. It is worth remembering that ferromagnetism was observed in the layered FeSe·(Li,Fe)(OH) together with superconductivity.

Valleriite and tochilinite appear to be narrow-gap semiconductors, with their electronic properties mostly determined by the metal sulfide layers. It is likely that a wider gap or metallic conductivity can be obtained by varying the composition of the chalcogenide part; however, experimental studies of conductivity and other physical parameters are still absent. Again, we leave aside FeSe-based superconductors and a potential superconductivity in valleriites, although the independent control of the layer characters paves new ways in this direction.

Fascinating optical properties were found in valleriite and tochilinite nanoflakes, particularly an intense absorption in the visible region that is likely due to all-dielectric resonances in submicrometer particles, which are of great interest for new-generation nanophotonics and solar energy conversion, photocatalysis and sensors. The additional absorption maxima below 500 nm belonging to Fe-S charge transfer suggest a ladder of Fe 3d states in the fundamental gap akin to Fe-S clusters in proteins, which can be promising in catalysis, photo- and electrocatalysis, electrochemical energy storage and so forth.

A few available studies aimed at examination of reactivity of valleriites have demonstrated, first of all, their relative stability in ambient air, aqueous media (not acidic) and under heating, especially in inert atmosphere. The interesting effect of impeded heat transport across the molecular layers (but not in the plane) as a function of concentration of Fe in the hydroxide sheets was found.

In general, the little research conducted on these type of materials was mainly directed either on the problems of mineral origination on Earth and in meteorites, or on superconductivity. The studies were restricted within the methods and chemical compositions of the materials of interest; to the best of our knowledge, no theoretical simulation of valleriites has been performed yet (if not mentioning the FeSe-related superconductors). Consequently, we tried to demonstrate in this review that the 2D heterolayered chalcogenide–hydroxide materials are a big new class of solid substances with unique characteristics, many of which are still waiting to be discovered, explored and utilized.

## Figures and Tables

**Table 1:** Summary of hydrothermal syntheses of heterolayered materials.

Products of Synthesis	Starting Materials	Reaction Conditions	Reference(s)
Valleriite, chalcopyrite, brucite, pyrrhotite, covellite, boehmite	CuFeS_2_, MgO, γ-Al_2_O_3_	1000 bar, 5–20 days, 400–700 °C	K. Iiishi et al. [87] (1970)
Selenian valleriite, valleriite, metal sulfides (selenides), korshunskite	CuFeSe_2_, CuFe_2_S_3_, MgO and Al_2_O_3_	1000 bar, 10–19 days, 450 °C	S. Takeno and G. Moh [88] (1994)
Valleriite as thin platy crystals (100–200 nm)	Fe^2+^/Cu^2+^, excessive (NH_4_)_2_S Mg^2+^/Al^3+^ + 25% NH_4_OH	pH 8.5–9.5, H_2_ pressure of 10 Bar, 1–25 days, 110–300 °C	A. Hughes et al. [89] (1993)
Valleriite (<50%), chalcopyrite, Fe (hydr)oxides	FeSO_4_, CuSO_4_ and MgSO_4_ solutions, H_2_S (or Na_2_S), NaOH	Varying ratio Cu:Fe:Mg 30 days, 150 °C, 180 °C	N.Chistyakova et al. [90,91,92] (2006), (2006), (2012)
Valleriite, valleriite doped with Al, Li, Ni, Cr, Co, La, 100–200 nm flakes 10–20 nm thick	Sulfates of Fe, Cu and doping elements, excessive Na_2_S, sulfates of Mg, Al, NH_4_OH	160 °C, 10–80 h, initial pH 9.5, final pH 12–12.5	Y. Mikhlin et al. [93] (2022)
Tochilinite as plate- and tube-like sub-μm particles	Aqueous Fe^2+^, excess of (NH_4_)_2_S (Mg,Al)-hydroxide gel, NH_4_OH	pH 8.5–9.5, H_2_ pressure of 2 MBar 2 days, 200 °C	G. Kakos et al. [94] (1994)
Fe-tochilinite, magnetite, mackinawite, pyrrhotite	Aqueous Fe oxyhydroxide, H_2_S, NaOH	pH 7.8 and 11.5, 30–150 days 80 °C	S. Kozerenko et al. [95] (1996)
Tochilinite, Fe-tochilinite, magnetite, mackinawite	Aqueous Fe^2+^, metallic Mg, NaOH, H_2_S	pH > 12 10–45 days 120–140 °C	L. Moroz et al. [96] (1997), S. Kozerenko et al. [97] (2001)
Tochilinite (low yield), magnetite, troilite and pyrite	Fe(OH)_2_, H_2_S, Mg	Medium-alkaline no data of synthesis times 160–180 °C	N. Chistyakova, T. Gubaidulina et al. [90,91] (2006, 2007)
Tochilinite (<40%) in the mixtures characteristic of meteorites	FeMgAl alloy particles, aqueous solutions of Na_2_S or (NH_4_)_2_S, or S^0^, NaOH	N_2_ or Ar atmosphere, pH 13–14, 4–120 (typically 40–60) days, 105–160 °C	Y. Peng et al. [98,99,100] (2007, 2009, 2014)
Mg,Fe-Tochilinite, Al,Fe-tochilinite, Na-tochilinite, sub-μm platelets few nm thick, nanotubes	Elemental Fe and S (Fe/S ≥ 1), Al, Mg, Na hydroxides	3–4 days, H_2_ 130–160 °C	R. Bolney et al. [101] (2022)
Tochilinite, Al-, Li-doped, flakes ~150 nm and 10–20 nm thick	aqueous sulfates of Fe, Mg, Al, Na_2_S, NH_4_OH	pH after synthesis 12–12.5 40 h, 160 °C	Y. Mikhlin et al. [103] (2023)
FeSe·(Li,Fe)OH powder (μm crystals)	Fe metal powder, excess of selenourea, LiOH	3–10 days 160 °C	X.F. Lu [107] (2015)
FeS·(Li,Fe)OH, powder of ~10–50 μm	Fe metal powder, thiourea, LiOH	3 days, 200 °C	X Zhang et al. [111] (2015)
FeSe·(Li,Fe)OH, 1–2 cm single crystals	A_x_Fe_2−y_Se_2_ (A = K, Rb, Cs) single crystal precursors, Fe metal powder, selenourea, LiOH	2 days 120–200 °C	X. Dong et al. [115] (2015), G. Yu et al. [116] (2016)

## Data Availability

No new data were created or analyzed in this study. Data sharing is not applicable to this article.
